# Leveraging metabolism for better outcomes in heart failure

**DOI:** 10.1093/cvr/cvae216

**Published:** 2024-10-01

**Authors:** Yann Huey Ng, Yen Chin Koay, Francine Z Marques, David M Kaye, John F O’Sullivan

**Affiliations:** Cardiometabolic Medicine, School of Medical Sciences, Faculty of Medicine and Health, The University of Sydney, Room 3E71 D17, Camperdown, NSW 2006, Australia; Charles Perkins Centre, Faculty of Medicine and Health, The University of Sydney, Office 3E72, Camperdown, NSW 2006, Australia; Cardiometabolic Medicine, School of Medical Sciences, Faculty of Medicine and Health, The University of Sydney, Room 3E71 D17, Camperdown, NSW 2006, Australia; Charles Perkins Centre, Faculty of Medicine and Health, The University of Sydney, Office 3E72, Camperdown, NSW 2006, Australia; Hypertension Research Laboratory, School of Biological Sciences, Faculty of Science, Monash University, Melbourne, VIC 3800, Australia; Heart Failure Research Group, Baker Heart and Diabetes Institute, Melbourne, VIC 3800, Australia; Victorian Heart Institute, Monash University, Melbourne, VIC 3800, Australia; Heart Failure Research Group, Baker Heart and Diabetes Institute, Melbourne, VIC 3800, Australia; Department of Cardiology, Alfred Hospital, Melbourne, VIC 3004, Australia; Monash-Alfred-Baker Centre for Cardiovascular Research, Monash University, Melbourne, VIC 3800, Australia; Cardiometabolic Medicine, School of Medical Sciences, Faculty of Medicine and Health, The University of Sydney, Room 3E71 D17, Camperdown, NSW 2006, Australia; Charles Perkins Centre, Faculty of Medicine and Health, The University of Sydney, Office 3E72, Camperdown, NSW 2006, Australia; Department of Cardiology, Royal Prince Alfred Hospital, Camperdown, NSW 2050, Australia; Department of Medicine, Technische Univeristat Dresden, 01062 Dresden, Germany

**Keywords:** Heart failure, Cardiac metabolism, SGLT2i, Microbiome, Epigenetics, Therapy

## Abstract

Whilst metabolic inflexibility and substrate constraint have been observed in heart failure for many years, their exact causal role remains controversial. In parallel, many of our fundamental assumptions about cardiac fuel use are now being challenged like never before. For example, the emergence of sodium–glucose cotransporter 2 inhibitor therapy as one of the four ‘pillars’ of heart failure therapy is causing a revisit of metabolism as a key mechanism and therapeutic target in heart failure. Improvements in the field of cardiac metabolomics will lead to a far more granular understanding of the mechanisms underpinning normal and abnormal human cardiac fuel use, an appreciation of drug action, and novel therapeutic strategies. Technological advances and expanding biorepositories offer exciting opportunities to elucidate the novel aspects of these metabolic mechanisms. Methodologic advances include comprehensive and accurate substrate quantitation such as metabolomics and stable-isotope fluxomics, improved access to arterio-venous blood samples across the heart to determine fuel consumption and energy conversion, high quality cardiac tissue biopsies, biochemical analytics, and informatics. Pairing these technologies with recent discoveries in epigenetic regulation, mitochondrial dynamics, and organ-microbiome metabolic crosstalk will garner critical mechanistic insights in heart failure. In this state-of-the-art review, we focus on new metabolic insights, with an eye on emerging metabolic strategies for heart failure. Our synthesis of the field will be valuable for a diverse audience with an interest in cardiac metabolism.

## 1. Introduction

The study of cardiac metabolism has its origins in the frog heart, followed by the development of the Langendorff isolated heart perfusion, the use of calorimetry, and the identification of sugar and fat use by the heart.^1^ Richard John Bing made the first inroads to the understanding of human myocardial metabolism in normal^2^ and diseased hearts.^3^ As then, critical advances in our understanding have included the study of hypoxia, glucose metabolism, anaerobic glycolysis, the relationship between pressure loading and self-renewal at transcriptional, translational, and post-translational levels, along with an improved understanding of mitochondrial function in obesity and diabetes.^4^ Recently, the application of multi-omic technology to transcardiac arterio-venous sampling has revealed novel insights into the relationship of left ventricular mechanical work to bioenergetics in normally functioning and failing hearts.^5,6^

Unfortunately, pertinent insights into cardiac metabolism in basic science or related clinical fields such as endocrinology are often outside the attention of the cardiology mainstream. This review attempts to summarize for a cardiology audience the key recent advances in the field, their relevance for the field of heart failure (HF), and their impact on future therapies. To enable comprehension of a complex topic, we have synthesized the literature into two overarching sections—The Problem and The Solution—with subsections in each highlighting the most important issues.

## 2. The problem

### 2.1 Act I—‘a broken switch’

#### 2.1.1 Substrate utilisation in the healthy heart

**Figure 1:**
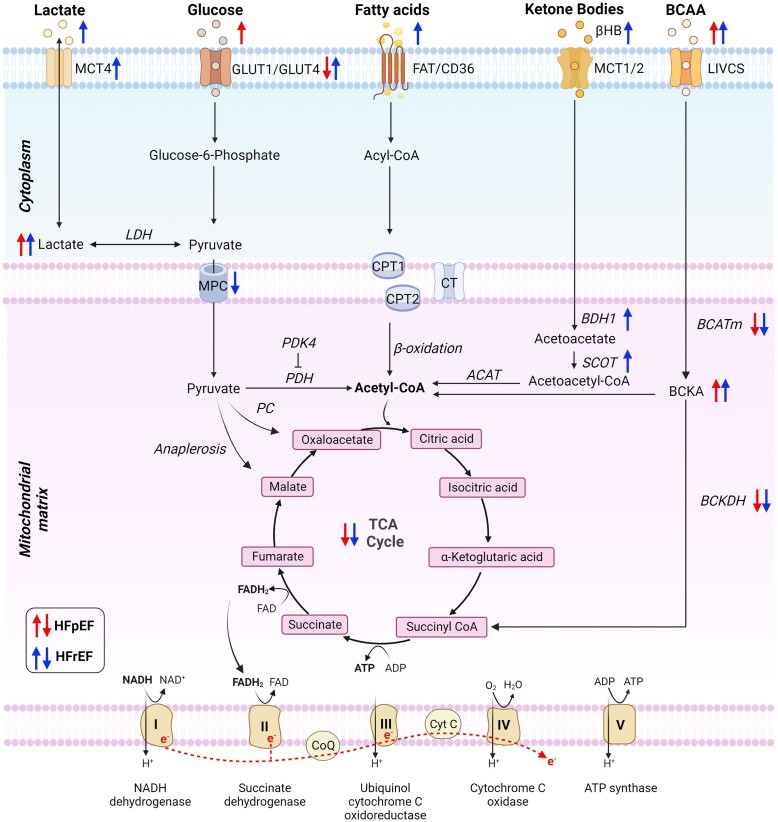
Cardiac energy metabolism in the healthy vs. failing heart and mitochondrial electron transport chain. Red arrows indicate changes in HFpEF whereas blue arrows indicate changes in HFrEF. ACAT, acetyl-CoA acetyltransferase; ADP, adenosine diphosphate; ATP, adenosine triphosphate; BCATm, mitochondrial branched-chain aminotransferase; BCKA, branched-chain ketoacids; BCKDH, branched-chain α-ketoacid dehydrogenase; βHB, β-hydroxybutyrate; BDH1, β-hydroxybutyrate dehydrogenase 1; CD36, cluster of differentiation 36; CoQ, coenzyme Q; CPT1, carnitine palmitoyltransferase-1; CPT2, carnitine palmitoyltransferase-2; CT, carnitine-acylcarnitine translocase; Cyt C, cytochrome C; FAD, oxidized flavin adenine dinucleotide; FADH_2_, reduced flavin adenine dinucleotide; FAT, fatty acid translocase; GLUT1, glucose transporter 1; GLUT4, glucose transporter 4; LDH, lactate dehydrogenase; LIVCS, branched-chain amino acid:cation symporter family; MCT1, monocarboxylase pyruvate transporter 1; MCT4, monocarboxylate transporter 4; MPC, mitochondrial pyruvate carrier; NAD^+^, nicotinamide adenine dinucleotide; NADH, nicotinamide adenine dinucleotide hydrogen; PC, pyruvate carboxylase; PDH, pyruvate dehydrogenase; PDK4, pyruvate dehydrogenase kinase isoenzyme 4; SCOT, succinyl-CoA:3 oxoacid-CoA transferase; TCA, tricarboxylic acid.

Cardiac energy is generated mostly from the oxidation of long-chain fatty acids (LCFAs, >12 carbons). LCFAs enter the cells via fatty acid translocase/cluster of differentiation 36 (FAT/CD36) and are esterified to form fatty acyl-coenzyme A (CoA), which is then transported by carnitine palmitoyltransferase (CPT) into the mitochondria (*Figure 1*).^7^ Medium-chain (MCFAs, 6–12 carbons) and short-chain fatty acids (SCFAs, <6 carbons) enter the cell either via the monocarboxylate transporter 1 (MCT1), CPT1, or freely diffuse into the mitochondria. In the mitochondria, all fatty acids undergo β-oxidation to form acetyl-CoA that enters the tricarboxylic acid (TCA) cycle.

Myocardial glucose uptake is driven by insulin-independent glucose transporters (GLUT1) or insulin-sensitive GLUT4. Most glucose undergoes glycolysis producing either pyruvate or is catalysed by lactate dehydrogenase (LDH) into lactate in anaerobic conditions. Conversion of glucose to lactate via anaerobic glycolysis yields two ATP molecules per glucose molecule and does not require oxygen. Under aerobic conditions, only about 3%–6% of pyruvate in the heart is converted to lactate,^8^ the rest is transported into the mitochondria and decarboxylated to acetyl-CoA, which enters the TCA cycle. Pyruvate can also undergo carboxylation to form oxaloacetate or be catalysed by malic enzyme to form malate to replenish TCA intermediates (anaplerosis). Additionally, lactate is constantly taken up by the heart via the MCTs both at rest and with increased workload,^9^ contributing up to 2.8% of ATP in the human heart during the fasted state.^6^ The reciprocal relationship between fatty acid and glucose utilisation where a decrease in fatty acid oxidation (FAO) causes an increase in glucose oxidation and vice-versa, is termed the Randle cycle (or glucose–fatty acid cycle).

Ketone bodies (i.e. β-hydroxybutyrate and acetoacetate) are taken up by the heart via MCT1 or MCT2. In the mitochondria, β-hydroxybutyrate is catalysed by β-hydroxybutyrate dehydrogenase 1 (BDH1) to acetoacetate, which is then converted to acetoacetyl-CoA and subsequently to acetyl-CoA by succinyl-CoA:3 oxoacid-CoA transferase (SCOT) and acetyl-CoA acetyltransferase (ACAT), respectively.

Branched-chain amino acids (BCAAs) (i.e. leucine, isoleucine, and valine) are transported into the cell by branched-chain amino acid:cation symporter family (LIVCS). In the mitochondria, BCAAs undergo reversible transamination, producing branched-chain α-keto acids (BCKAs), which are then oxidized to form either acetyl-CoA for the TCA cycle or succinyl-CoA for anaplerosis. BCAAs account for <2% of ATP production in the heart.^6^

FAO yields the greatest amount of ATP (105 mol ATP/mol palmitate) when compared to other substrates (31 mol ATP/mol glucose; 20 mol ATP/mol ketones).^10^ However, the oxygen demand is the highest for fatty acids as the heart consumes 23 molecules of O_2_ per palmitate molecule with a phosphate/oxygen [P/O] ratio of 2.33 when compared to glucose (P/O = 2.58) and β-hydroxybutyrate (P/O = 2.50).^10^

#### 2.1.2 Metabolic remodelling in the failing heart

The concept that the failing heart is metabolically inflexible and unable to switch amongst different energy substrates to fulfil its metabolic demands is mainly derived from animal studies and may not represent the failing human heart. In patients with non-ischaemic heart failure with reduced ejection fraction (HFrEF), intra-venous infusion of insulin–glucose at rest increased cardiac glucose utilisation, whereas supply of free fatty acids by Intra-lipid infusion elevated fatty acid utilisation.^11^ Additionally, insulin–glucose infusion during increased cardiac workload induced by rapid atrial pacing increased LCFA uptake and oxidation.^11^ These results suggest that the failing heart has active substrate selection in response to acute exogenous administration of substrate and preference to fatty acids over glucose during increased workload. This study challenges the current notion that metabolic flexibility is impaired in the failing heart. Nevertheless, myocardial phosphocreatine (PCr) to ATP ratio and ATP level are consistently reduced in both HFrEF^12^ and heart failure with preserved ejection fraction (HFpEF) patients.^13^ Disrupted metabolic homeostasis is closely coupled to cardiac dysfunction in HF and is postulated as the proximal driver.

The concept that the failing heart is an ‘engine out of fuel’ is decades old.^14^ Recently, Rodolico *et al.* pointed out that the failing myocardium will never run out of fuel when oxygen supply is sufficient, and that there is fuel overload in patients at risk of HF.^15^ Obesity and diabetes are independent risk factors associated with the development of HF. Obesity was associated with cardiac steatosis, and was an independent predictor of increased myocardial oxygen consumption and reduced myocardial efficiency.^16^ Myocardial lipid deposition was increased in participants with insulin resistance prior to the onset of diabetes,^17^ HFrEF,^18^ and HFpEF patients.^13^ These studies suggest that substrate availability in the failing heart is not depleted; instead, there is impaired capacity to utilize substrate with spillover in the form of lipid storage and concomitant reduction in myocardial efficiency.

Whilst a decrease in fatty acid metabolism is frequently observed in HFrEF, changes in fatty acid metabolism are inconsistent in HFpEF. Myocardial fatty acid uptake was unaltered at an early stage of compensated left ventricular hypertrophy but reduced in a rodent model with severe congestive HF.^19^ Fatty acid uptake rather than oxidation was impaired in the myocardium of patients with end-stage, non-ischaemic dilated cardiomyopathy (DCM).^20^ Acute delivery of fatty acids via intra-venous infusion increased fatty acid utilisation and improved cardiac bioenergetics in non-ischaemic HFrEF patients.^11^ These studies suggest that fatty acid uptake and oxidation is dependent on the disease stage, with little impairment of both in the early stages of HF, but severe impairment of uptake with unaltered oxidation in advanced stages of non-ischaemic cardiomyopathy. Whilst acute elevation of fatty acid concentration may be beneficial, further studies are required to determine whether the degree of functional impairment or aetiology of HF would affect this response, whether this response is maintained over time, and to what extent it can be delivered without spillover, storage, or lipotoxicity.

HF is often characterized by an increased reliance on glucose. In HFrEF patients with DCM, myocardial glucose uptake and glycolysis were increased.^21^ Surprisingly, there was almost no glucose uptake by the heart in HFrEF patients in the fasted state, which could be due to a response to fasting.^6^ Increased glucose flux into glycolysis and anaplerosis in the TCA cycle increased aspartate synthesis and promoted cardiac hypertrophy in rat cardiomyocytes,^22^ suggesting that increased glycolysis may promote pathological hypertrophic responses in HF. Unlike HFrEF, *SLC2A1* (solute carrier family 2 member 1) gene, which encodes GLUT1 was lower, but mitochondria pyruvate carriers, MPC1 was up-regulated in HFpEF patients.^23^ However, both MPC1 and MPC2 were down-regulated in end-stage advanced HFrEF patients with DCM.^24^ These data suggest suppressed mitochondrial pyruvate oxidation in HFrEF but not in HFpEF. Enhancing glucose oxidation by intra-coronary infusion of pyruvate^25^ or administration of dichloroacetate (a PDK inhibitor)^19^ improved cardiac function in HF patients, suggesting that restoring glucose oxidation may be beneficial.

Increased glycolysis and reduced glucose oxidation in the failing heart may suggest that pyruvate is being converted to lactate in the cytosol instead of being oxidized in the mitochondria. Myocardial lactate consumption increased from 2.8% (in non-failing heart) to 5% for ATP generation in HFrEF patients in the fasted state.^6^ In older HFpEF patients (≥80 years of age), the expression of LDHB, which catalyses the conversion of lactate to pyruvate was reduced, and plasma lactate level was increased during acute decompensation.^26^ High blood lactate levels were associated with increased risk of mortality and poor prognosis in patients with acute decompensated HFrEF^27^ and cardiogenic shock.^28^ Inhibition of MCT4 (cellular lactate exporter) prevented cellular hypertrophy in cardiomyocytes and in a mouse model with cardiac hypertrophy, potentially by redirecting glycolytic carbon flux back towards mitochondrial pyruvate oxidation.^24^ At present, the mechanism of lactate oxidation in HF remains incompletely understood, and elevated plasma lactate level may be a potential biomarker to predict HF prognosis.

Circulating blood ketone bodies^29^ and myocardial ketone body utilisation^6^ are reportedly increased in HFrEF patients. Increased β-hydroxybutyrate uptake during ketosis was correlated to left ventricular remodelling and left ventricular mass, and inversely correlated to ejection fraction in chronic HFrEF patients.^30^ Conversely, increasing circulating ketone bodies acutely via either intra-venous infusion of β-hydroxybutyrate or a single-dose enteral treatment with ketone ester improved cardiac function in patients with HFrEF,^31^ cardiogenic shock,^32^ and pulmonary hypertension.^33^ A recent study also showed that oral administration of ketone ester in patients with stable HFrEF four times daily for 14 days improved resting cardiac output and ejection fraction, and reduced pulmonary capillary wedge pressure (lower filling pressure) and NT-proBNP levels,^34^ suggesting the potential therapeutic effects of ketone bodies in HF. However, due to a lack of clinical data in HFpEF, further studies are required to determine whether increasing ketone bodies is beneficial for the treatment of HFpEF.

Animal and human data show that cardiac BCAA and BCKA levels are elevated, and gene expression of enzymes involved in BCAA metabolism is reduced in both HFrEF and HFpEF, suggesting that myocardial BCAA oxidation is impaired in the failing heart.^23,35^ However, using *in vivo* isotope infusions, cardiac BCAA oxidation was increased rather than suppressed in a murine model of HFrEF.^35^ Direct assessment of BCAA oxidation in HF patients is lacking. Accumulation of BCAA and BCKA in the failing heart due to impaired BCAA oxidation may induce mitochondrial oxidative stress,^36^ promote insulin resistance,^37^ and induce cardiac hypertrophy via chronic activation of mTOR.^38^ Stimulating BCAA catabolism improved cardiac function in DCM patients,^36,37^ suggesting that improving BCAA oxidation may be beneficial for HF treatment.

### 2.2 Act 2—‘a damaged furnace’

#### 2.2.1 The impact of metabolic dysfunction on cardiac mitochondrial dynamics

Aristotle considered the heart to be the body’s furnace, radiating heat to support the body’s energy demands,^39^ but the true furnace is arguably the mitochondrion within the cardiomyocyte. The heart contains the highest content of mitochondria of any tissue.^40^ This is required to meet the heart’s enormous energy requirements to sustain unrelenting mechanical work, as both contraction and relaxation are active processes requiring energy. In the previous section, having outlined how the heart has a substantial capacity to utilize different substrates to generate ATP under normal and disease states, we will now discuss how mitochondrial function is a critical mediator of these processes. We will focus on bioenergetics; other potential mechanistic roles of mitochondria in HF such as in oxidative stress, mitochondrial fission, and mitochondrial calcium regulation are outside the scope of this review.^41^ We focus on human data, but also discuss pre-clinical data that we believe advances mechanistic understanding of human pathological insights.

Previous attempts to address mitochondrial generation of ATP and FAO have not yielded clinically impactful and safe outcomes. For example, perhexiline, a CPT1 inhibitor, increased the PCr/ATP ratio and improved New York Heart Association (NYHA) functional class, but did not improve left ventricular ejection fraction or cardiac substrate utilisation in DCM patients.^42^ Perhexiline administration is also associated with hepatic and neurological adverse effects, which limit its use for HF therapy.^43^ In addition, a clinical trial involving the administration of etomoxir, another CPT1 inhibitor, had to be stopped pre-maturely due to the occurrence of hepatoxicity.^44^ Trimetazidine, an inhibitor of long-chain3-ketoacyl-CoA thiolase reduces HF hospitalisation and improves NYHA functional class and ejection fraction, but its effect on PCr/ATP is controversial.^45,46^

Nonetheless, the impetus for understanding the therapeutic potential offered by mitochondria has been reaffirmed with the emergence of HF pharmacotherapy with, perhaps serendipitous, effects on mitochondrial function. All previous HF therapies acted to reduce the workload on the failing heart and, in doing so, reset the heart’s energy demand threshold to a lower level.^47^ Although not addressing the energy imbalance, these therapies have favourable effects on deleterious cardiac remodelling. However, the reset to a lower energetic demand ignores the energetic ‘supply’ side of the equation and does not directly address the impaired bioenergetic capacity of the heart.^48^ Indeed, these therapies that systemically block maladaptive neurohormonal perturbation also lower blood pressure and development of newer drugs for stepwise addition to these medications raise tolerability and safety concerns with regard to compounding hypotension and/or electrolyte disturbance.^47^ Therefore, an ideal novel therapy targeting myocardial bioenergetics would be hemodynamically neutral.

Ketone pathways are a potential target to improve mitochondrial function in HF. Mitochondrial protein hyperacetylation was associated with a deficiency of nicotinamide adenine dinucleotide (NAD^+^) and impaired mitochondrial FAO in a 2-hit HFpEF murine model induced by a high-fat diet and L-NAME.^49^ In a 3-hit HFpEF murine model induced by aging, high-fat diet and desoxycorticosterone pivalate, elevation of β-hydroxybutyrate reduced mitochondrial protein acetylation, inflammation, cardiac brain natriuretic peptide (BNP) level, and attenuated cardiac stiffness.^50^ Elevating NAD^+^ by oral supplementation of nicotinamide riboside^49,51^ or nicotinamide^52^ improved mitochondria respiration and rescued HFpEF in murine models. NAD^+^ repletion also elevates cardiomyocyte ketogenic enzymes and ketone levels (YC Koay, unpublished work). Thereby, a promising inter-section of NAD^+^ repletion, increased mitochondrial function, and ketogenic pathway signalling warrants dedicated study as a promising therapeutic realm in the mitochondrial biology of HF.

### 2.3 Act 3—‘*post hoc* governors’

#### 2.3.1 DNA (CpG) methylation

**Figure 2:**
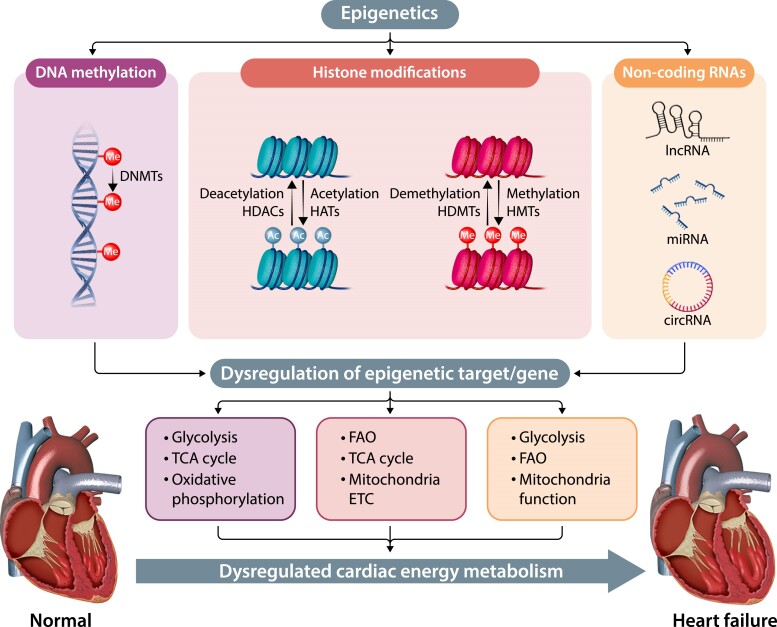
The epigenome: an important player in the regulation of cardiac metabolism in heart failure. circRNA, circular RNA; DNMTs, DNA methyltransferases; ETC, electron transport chain; FAO, fatty acid oxidation; HATs, histone acetyltransferases; HDACs, histone deacetylases; HDMTs, histone demethylases; HMTs, histone methyltransferases; lncRNA, long non-coding RNA; miRNA, microRNA; TCA, tricarboxylic acid.

In recent years, there has been a significant focus on understanding the epigenetic mechanisms linked to cardiac hypertrophy and HF pathophysiology, including DNA methylation, post-translational modifications of histones and non-coding RNAs (ncRNAs). These modifications (summarised in *Figure 2*) can affect gene expression by altering chromatin structure, which in turn affects DNA accessibility and gene transcription in relation to energy metabolism in the failing hearts.

DNA methylation of cytosine at the 5′ position of pyrimidine (CpG methylation), catalysed by DNA methyltransferases (DNMT), helps regulate epigenetic gene expression,^53^ with promoter hypermethylation decreasing gene expression and promoter hypomethylation leading to an increase in gene expression. Notably, *de novo* methylation is primarily regulated by DNMT3A and DNMT3B in the heart.^54^ DNMT3A knockout in engineered heart tissue generated from human induced pluripotent stem cell-derived cardiomyocytes impaired mitochondrial cristae structure and glucose metabolism, reduced respiration rate and ATP production, and altered contraction kinetics.^55,56^ Deletion of DNMT3B in mice compromised heart function under normal conditions and worsened HF during hypertrophy-inducing stimuli such as pressure overload induced by transverse aortic constriction (TAC) or isoproterenol.^57^ Genomic regions are also differentially methylated during pre-natal development and post-natal maturation in human hearts.^58^ These studies show the important role of DNA methylation in regulating mitochondrial function, cardiac development, metabolism, energy output, and the heart's response to stress.

DNA methylation potentiates alteration in cardiac gene expression in HF. Left ventricular tissue from end-stage HFrEF patients exhibited hypomethylation and induction of glycolytic enzymes (i.e. phosphofructokinase, enolase) as well as hypermethylation and silencing of genes involved in TCA (i.e. succinate dehydrogenase), oxidative phosphorylation [i.e. cytochrome c oxidase (*COX17*)], and FAO (i.e. acetyl-CoA acyltransferase).^59^ Furthermore, HF aetiology-specific changes in DNA methylation were identified in inter-ventricular septal tissues obtained from patients with ischaemic cardiomyopathy, DCM, and hypertrophic obstructive cardiomyopathy (HOCM) relative to matched non-failing donor hearts.^60^ In this study, *MSR1* (*macrophage scavenger receptor 1*, involved in inflammatory response and lipid homeostasis) was hypermethylated in HOCM biopsies; *MMP2* (*matrix metallopeptidase 2*, a regulator of collagen turnover and fibrosis) and *CTGF* (*connective tissue growth factor*, involved in fibrosis) were hypomethylated in DCM tissues; whereas *MYOM3* (*myomesin 3*, involved in muscle contractility and myofibril assembly) and *COX17* were hypermethylated in ischaemic cardiomyopathy biopsies. These findings highlight the significant impact of DNA methylation on cardiac metabolism, HF development and aetiology, and suggest that DNA methylation may be a potential therapeutic target for HF.

#### 2.3.2 Histone modifications

##### 2.3.2.1 Histone acetylation and deacetylation

Acetylation typically occurs on the lysine residues of histone, and is facilitated by histone acetyltransferases (HATs, ‘writer’) and histone deacetylases (HDACs, ‘eraser’). There are two classes of HATs: type A HATs are localized in the nucleus and involved in chromatin remodelling and nucleosomal histone modifications whereas type B HATs are located in the cytoplasm and facilitate nucleosome formation.^61^ HDAC are categorized into Class I (HDAC1, 2, 3, and 8), Class IIa (HDAC4, 5, 7, and 9), Class IIb (HDAC6 and 10), Class III HDAC (NAD + -dependent sirtuins 1–7), and Class IV (HDAC11).

Changes in pathological gene expression are accompanied by alterations in active histone marks (i.e. H3K9ac, H3K27ac, H3K4me3, and H3K36me3) as well as repressive marks (i.e. H3K27me3) in the failing human hearts.^62^ Hyperacetylation of histone 3 acetylated at lysine 9 (H3K9) is associated with cardiac hypertrophy.^63^ Reduced H3K9 acetylation by inhibiting HAT activity of p300 attenuated hypertrophy and improved cardiac function in TAC-induced HFrEF mice.^63^ Furthermore, HDAC3 expression was reduced in peripheral blood mononuclear cells (PBMCs) isolated from coronary artery disease patients.^64^ Cardiac-specific deletion of HDAC3 in mice promoted cardiac hypertrophy, and this was associated with down-regulation of GLUT4, up-regulation of genes associated with FAO, and reduced mitochondria complex I activity.^65^ In contrast, HDAC6 protein expression was increased in TAC mice.^66^ HDAC6 deletion in mice rescued diastolic dysfunction and alleviated signs of HF.^66^ These studies suggest that epigenetic regulation is site specific and have different impacts towards the development of HF.

Alterations in Class III HDACs impair cardiac metabolism and contribute to HF. Sirt1 activity was higher in PBMCs isolated from HFrEF patients, but not different in HFpEF patients when compared to the non-HF controls.^67^ In contrast, Sirt1 expression was reduced in myocardial tissues of patients with advanced HF^68^ and type 2 diabetes.^69^ In *db/db* mice, treatment with recombinant Sirt1 rescued diabetes-induced cardiac dysfunction and this was associated with down-regulation of genes involved in lipid metabolism [i.e. *CD36*, *Pparg* (peroxisome proliferator-activated receptor γ)].^69^ Additionally, Sirt6 protein expression was reduced in the failing human hearts vs. non-failing controls.^70^ Administration of Sirt6 agonist MDL-800 in a pressure overload HF mouse model attenuated hyperacetylation of H3K9, alleviated mitochondrial ultrastructure damage, improved mitochondrial respiratory and cardiac function.^71^ Therefore, Class III HDAC may be a potential therapeutic target for HF.

##### 2.3.2.2 Histone methylation and demethylation

Histone methylation is regulated by lysine methyltransferases (KMTs) and demethylases (KDMs). Alterations in histone methylation are closely linked to HF. Histone H3 lysine 4 trimethylation (H3K4me3, an active promoter mark of gene activation) was decreased in left ventricular tissues from DCM^72^ and ischaemic cardiomyopathy patients with reduced ejection fraction.^73^ Reduced H3K4 methylation was associated with down-regulation of SMYD1, which is a H3K4-specific lysine methyl transferase that is essential for heart development and regulates cardiac mitochondrial energetics.^74^ In left ventricular tissues from patients with end-stage ischaemic and idiopathic cardiomyopathy, histone 3 lysine 36 trimethylation (H3K36me3) was enriched in active transcriptional regions of the genome, including protein-coding genes and ncRNA, and regulated the expression of double homeobox 4 (*DUX4*), which involved in early human embryo development.^75^ SET domain containing-2 (SETD2), a H3K36-specific trimethyltransferase was up-regulated in myocardial tissues obtained from obese HFpEF patients and this was correlated with cardiomyocyte stiffness.^76^ In the same study, cardiomyocyte-specific deletion of SETD2 in mice prevented hypertrophic remodelling and diastolic dysfunction. These findings suggest the involvement of histone methylation in HF pathogenesis.

Histone lysine demethylation is catalysed by lysine-specific demethylases (LSD) and Jumonji C domain-containing proteins (JMJD), which requires flavin adenine dinucleotide (FAD) and α-ketoglutarate as cofactors. The JMJD family such as JMJD2 and JMJD3 are α-ketoglutarate-dependent histone demethylases,^77^ and are sensitive to changes in TCA cycle intermediates. Under hypoxic conditions, the accumulation of metabolites such as 2-hydroglutaric acid, succinate, and fumarate inhibits JMJD activity, leading to global histone methylation changes.^78^ In contrast, increasing FAD production activates lysine-specific demethylase 1 enzyme (LSD1) and maintains phospholipid homeostasis is protective against ischaemia.^79^ Therefore, metabolic changes affect epigenetic regulation and contribute to HF.

Regulation of H3K36 demethylation by lysine demethylase 8 (KDM8) is critical for maintaining cardiac function by influencing mitochondrial function and energy metabolism. KDM8 maintains an active mitochondrial gene network by repressing TBX15, thus preventing DCM and lethal HF.^80^ KDM8 was down-regulated in human hearts affected by DCM, and higher TBX15 expression was correlated with the down-regulation of genes encoding mitochondrial proteins, suggesting that epigenetic dysregulation of metabolic gene networks, mediated by the KDM8-TBX15 axis, could initiate myocardial deterioration towards HF.^80^

#### 2.3.3 Non-coding RNA

ncRNAs are functional RNA molecules that are not translated into proteins, but act as regulators for epigenetics and gene expression. ncRNA has attracted increasing attention in HF, and can be broadly classified into small [i.e. microRNAs (miRNAs)] or long ncRNAs [i.e. linear long ncRNAs (lncRNAs), circular RNAs (circRNAs)]. Here, we will discuss the implications of ncRNA on cardiac metabolism and mitochondrial function in the context of HF with a focus on human data as the role of ncRNAs has been extensively described elsewhere.^81^

Alteration in ncRNAs expression affects cardiac energy metabolism and mitochondrial function in HF patients. For instance, miR-122-5p expression was increased in patients with aortic valve stenosis^82^ and acute myocardial infarction.^83^ Bioinformatic analysis revealed that miR-122-5p was closely related to glucose metabolism pathways including gluconeogenesis and glycolytic pathways in diabetic patients.^84^ Deletion of miR-122-5p impaired fatty acid metabolism in mice.^85^ miRNAs may also be used as potential biomarkers to distinguish between HFpEF and HFrEF patients, where a combination of miRNAs including miR-107, miR-139-5p, and miR-150-5p discriminated HFpEF from HFrEF.^86^ Furthermore, differentially expressed lncRNAs (i.e. AK055347) in epicardial adipose samples obtained from patients with atrial fibrillation vs. sinus rhythm,^87^ and between left atrial from the pulmonary vein vs. left atrial appendage in atrial fibrillation patients^88^ were associated with mitochondrial energy production, lipid metabolism, and stress response.

A recent study involving participants from the Helsinki Birth Cohort Study showed that increased maternal body mass index (BMI) was associated with elevated offspring serum miR-15b in humans.^89^ In the same study, overexpression of miR-15b-5p in embryonic rat cardiomyoblasts H9c2 cells impaired mitochondrial function and reduced FAO, suggesting that miR-15-b may contribute to cardiac metabolism dysregulation. Additionally, miRNA-132-3p was up-regulated in cardiac tissue in response to hypertrophic stimuli, which contributes to the development of HF.^90^ Overexpression of miRNA-132 down-regulated mitochondrial carnitine acyl-carnitine translocase, leading to the inhibition of β-oxidation,^91^ suggesting that increased miRNA-132 level is detrimental. Pre-clinical investigation in a porcine model of chronic HF showed that CDR132L, a miR-132 inhibitor improved cardiac function, and reversed cardiac remodelling.^92^ These studies highlight the role of ncRNAs in regulating cardiac metabolism and suggest that ncRNAs are promising therapeutic targets for HF.

#### 2.3.4 *Simul*—‘fermented disequilibrium’

**Figure 3:**
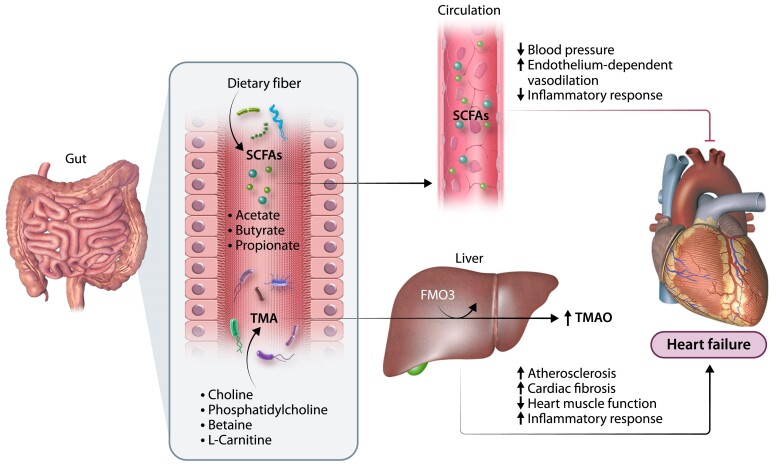
The inter-play between dietary metabolism, the gut microbiome, and heart failure. Key metabolic pathways include the microbiota-mediated fermentation of indigestible fibre into SCFAs, known for their protective effects such as reducing inflammation and improving vascular tone. On the other hand, diet-derived nutrients, like choline, phosphatidylcholine, betaine, and L-carnitine, can be metabolized by the gut microbiota into TMA. TMA is then converted in the liver into TMAO via the FMO family of enzymes. High levels of TMAO have been associated with atherosclerosis development, inflammation, cardiac fibrosis, and cardiac dysfunction, thereby increasing the risk of heart failure.

The microorganisms that inhabit the gastrointestinal tract, collectively known as the gut microbiota, contribute significantly to the metabolites found in the human circulation. Several microbiota-produced metabolites affect cardiac function as summarized in *Figure 3*.

##### 2.3.4.1 The gut microbiome and heart failure

It is relevant to note that there is a substantial change in the composition of gut microbiota of patients with HFpEF relative to healthy referents—these differences were independent of traditional risk factors such as age, sex, BMI, prevalence of hypertension, and diet.^93^ These changes were characterized by lower α-diversity (i.e. number and abundance of microbes) and abundance of *Ruminococcus* species, an important producer of SCFAs, amongst others.^93^ The clear separation between the microbiota of controls and cases, lower α-diversity and reduced prevalence of SCFA-producers have been also reported in patients with HFrEF.^94^

##### 2.3.4.2 Gut permeability

An important phenomenon that may be present in HF patients is an increased permeability of the gut epithelial barrier, which allows the translocation of microbes and/or their products to the host’s systemic circulation, eliciting inflammation.^95^ Markers of gut permeability (e.g. zonulin, intestinal fatty acid binding protein, lipopolysaccharide-binding protein) have been reported in patients with hypertension^96^ and with atrial fibrillation.^97^ An *in vivo* assay was used to measure intestinal permeability in 29 patients with decompensated HF—those with higher permeability had a higher risk of adverse outcomes in the 56-days they were followed.^98^ Collectively, these studies suggest that the gut microbiota is altered in patients with HF and that the breakdown of the gut epithelial barrier may be observed before the development of HF, contributing to its development. Larger, multi-centre studies with well-phenotyped control participants are warranted to confirm these findings.

##### 2.3.4.3 Short-chain fatty acids

Lower intake of dietary fibre is associated with higher coronary heart disease incidence and mortality, with a dose-response relationship showing a benefit with more than ∼28 g of fibre consumed per day, especially with intake >35 g/day.^99^ This is of concern considering the global average intake of fibre is ∼11 g/day across low-, middle-, and high-income countries.^100^ Fibre reaches the large intestine undigested, where some types of fibre (e.g. resistant starches and some soluble fibres) are fermented by the gut microbiota.^101^ This fermentation leads to the release of SCFAs as by-products.^101^ The most abundantly released SCFAs are acetate, propionate, and butyrate—these have important inter-actions with the immune system,^102^ and priming^103^ and increasing the prevalence of anti-inflammatory immune cells such as T regulatory cells.^104^ Importantly, the heart is a major site of acetate uptake according to experiments using *in vivo*^11^C-acetate and positron emission tomography (PET)–computed tomography scanning.^105^ Indeed, supplementation with acetate in the drinking water for 3-weeks modified the cardiac transcriptome in mice, modifying pathways relevant to HF including down-regulation of extracellular matrix receptor and the renin–angiotensin system.^106^ Other mechanisms may involve SCFA sensing receptors, such as G-protein coupled receptors GPR41, GPR43, and GPR109A, as lack of sensing via one or more of these receptors causes cardiac dysfunction.^104^ These may act via the breakdown of the gut epithelial barrier, regulation of the immune system, or via epigenetic mechanisms such as inhibition of histone deacetylases.^95^

Although there is evidence that levels of dietary fibre intake are reduced in HFpEF patients (17.6 ± 7.7 g vs. 23.2 ± 8.8 g/day)^93^ and HF patients in general have lower levels of SCFA-producing bacteria,^93^ we are not aware of any direct evidence that levels of SCFAs are decreased in HF. However, interventions with SCFAs, even when fibre intake was low, reduced cardiac hypertrophy and fibrosis and improved cardiac function in deoxycorticosterone acetate/salt and angiotensin II experimental models.^106^ An opportunity may be the use of SCFA-enriched fibres such as acetylated and butyrylated high amylose maize starch (HAMSAB), as below.^107^ Together, this evidence highlights the potential for SCFAs to improve cardiac function of HF patients.

##### 2.3.4.4 Trimethylamine N-oxide (TMAO)

The most studied gut microbiota-derived metabolite that contributes to cardiovascular disease risk is TMAO.^108^ Dietary choline and L-carnitine, found in red meat, eggs, and dairy products resulted in higher microbial levels of TMAO’s precursor, trimethylamine (TMA).^108,109^ This phenomenon was not observed in germ-free mice, which lack microbiota.^108,109^ In the liver, TMA is converted to TMAO by the isoforms 1 and 3 of flavin-dependent monooxygenase (FMO). In a prospective study with 720 HF patients and 300 healthy referents, TMAO levels were higher in HF patients, as well as associated with higher mortality and BNP levels compared to those with normal heart function.^110^ In another study with 112 HF patients, TMAO was also associated with left ventricular diastolic dysfunction.^111^ Using a pressure-overload model of HF, choline and TMAO increased cardiac fibrosis, hypertrophy and reduced left ventricular ejection fraction relative to the control diet.^112^

Although L-carnitine is a precursor of TMAO, considerable studies have shown the benefits of L-carnitine supplementation in HF patients. Several meta-analyses of randomized controlled trials have revealed that L-carnitine supplementation is associated with improvement in left ventricular ejection fraction, cardiac output, and reduced BNP and NT-proBNP levels in patients with DCM^113^ and chronic HF.^114^ Similar improvement in ejection fraction has also been reported in patients with ischaemic HF undergoing coronary artery bypass graft surgery^115^ and in paediatric patients (<18 years of age) with impaired left ventricular function.^116^ In a rodent model of myocardial infarction, L-carnitine administration exerts cardiac protective effects by suppressing oxidative stress and increasing antioxidant enzyme functions.^117^ These studies suggest that L-carnitine supplementation may exert cardioprotective effects in HF.

In addition, there is some controversy regarding the role of TMAO in cardiovascular disease.^118^ Some studies have found that plasma TMAO levels were also increased with healthy diets including with intake of fish,^119^ low glycaemic load,^120^ and resistant starches.^121^ For example, in a small cross-over trial, a diet high in resistant starches, when combined with low carbohydrate intake, resulted in higher plasma TMAO levels relative to a diet low in resistant starch.^121^ These findings were replicated in high resistant starch-fed mice.^118^ This is likely explained by an increase in bile acids, which induce the expression of the *FMO* gene family.^118^

##### 2.3.4.5 Indole-3-propionic acid (IPA)

We have previously demonstrated in mice that high fibre dietary intervention dramatically increased plasma levels of IPA (22-fold),^122^ which is a gut-microbe derived metabolite of tryptophan. IPA levels were reduced in HFpEF and IPA supplementation reduced diastolic dysfunction in a HFpEF mouse model by promoting the expression of SIRT3 and restoring NAD^+^ levels.^123^ Although there is emerging evidence that IPA plays a role in cardiac contractility and hypertension,^124^ its mechanistic roles remain to be elucidated.

## 3. The solution

Given the critical role of metabolism in maintaining cardiac function, manipulating metabolic pathways has long been an unfulfilled target for improving heart function, although recent developments are showing promise. Here, we discuss various approaches to manipulating metabolism, including exercise, diet, drugs such as sodium–glucose cotransporter 2 inhibitor (SGLT2i) and glucagon-like peptide-1 receptor agonists (GLP-1RA), and potential new therapies such as gene and RNA-based interventions. We will examine the evidence for each approach and discuss their therapeutic implications for the prevention and treatment of HF.

### 3.1 Preventive metabolic strategies

The ACC/AHA 2022 Guidelines highlight Stage A HF as ‘at-risk for heart failure,’ which includes asymptomatic patients with readily identifiable risk factors including hypertension, diabetes, obesity, and existing cardiovascular disease, but without structural/functional heart disease or abnormal biomarkers; Stage B ‘pre-heart failure’ patients have evidence of structural heart disease, increased filling pressures, and/or increased BNP levels or persistently elevated cardiac troponin in the absence of competing diagnoses.^125^ These early stages afford a critical opportunity to start preventive strategies targeting metabolic health.

Although recent HF guidelines have included recognition of at-risk HF,^125^ further work is needed to increase awareness and institute early intervention. Cardiologists must be pro-active at investigating metabolic risk factors, including careful monitoring of glucose homeostasis. With the metabolic syndrome now present in >30% of adults and up to 50% of adults aged 60 or over,^126^ it is critical that interrogation of glucose homeostasis goes beyond fasting glucose or glycosylated haemoglobin (HbA1c) to include fasting insulin and oral glucose tolerance testing in overweight adults. Patients with metabolic syndrome have higher risk of developing type 2 diabetes, and early identification can prevent or delay diabetes and/or HF onset.

‘Metabolic unloading’ is a concept akin to unloading the heart from metabolic stress either via restriction of fuel supply or elimination of excess fuel.^127^ Weight loss is a critical subject to broach with at-risk patients. Dietary intervention for weight loss consists of several approaches including caloric restriction which entails a 30%–50% reduction in average daily energy intake^128^; time restriction involves a restriction on the daily eating window (i.e. intermittent fasting, time-restricted feeding); and dietary restriction of macronutrients in food (i.e. carbohydrate, protein, or fat) or specialized diets. Studies have shown that 16 weeks of caloric restriction^129^ or 12 weeks of 10-h restricted eating (*ad libitum* feeding from 0800 to 1800 and fasting between 1800 and 0800)^130^ reduced BMI, HbA1c level, and total cholesterol, triglycerides, and improved diastolic function in overweight/obese patients with type 2 diabetes. In HFpEF and HFrEF patients, caloric restriction (400–800 kcal deficit/day) induced weight loss, improved myocardial efficiency and lipid profiles,^131,132^ suggesting that weight loss may induce desirable effect on cardiac metabolism.

Specialized diets are impactful. The Mediterranean diet is characterized by a high consumption of fruits, vegetables, nuts, whole grains, and extra-virgin olive oil.^133^ Mediterranean diet has repeatedly demonstrated reduction in cardiovascular events,^134,135^ and was associated with improved cardiometabolic risk factors such as BMI, waist circumference, blood pressure, insulin resistance, and lipid profile in patients with metabolic syndrome.^136,137^ Long-term adherence to a Mediterranean diet, inversely associated with cardiometabolic disease risk, was associated with 40 microbial species, many of which are known SCFA producers such as *Faecalibacterium prausnitzii*, as well as an enrichment for microbial enzymes involved in fibre degradation and SCFA fermentation.^138^ However, in HF patients, adherence to the Mediterranean diet was not associated with a lower risk of all-cause and cardiovascular mortality.^139^ Clinical data are limited, therefore further clinical trials are required to elucidate the safety and the role of dietary interventions in patients with established HF.

Bariatric surgery is associated with reduced systolic blood pressure, HbA1c, low-density lipoprotein levels, left ventricular mass, and improved ejection fraction and global longitudinal strain,^140^ extending the notion of unloading to the metabolic realm. PET imaging revealed decreased myocardial fatty acid utilisation in obese participants after gastric bypass surgery,^141^ suggesting that bariatric surgery may affect myocardial substrate utilisation. In women with HFpEF, bariatric surgery improved symptoms, reversed cardiac remodelling, and improved diastolic function, but myocardial triglyceride was unchanged.^142^ Bariatric surgery was also associated with reduced risk of all-cause mortality and hospitalisation in HF patients.^143^ In terms of changes in the gut microbiome, type 2 diabetic patients after bariatric surgery had increased *F. prausnitzii* abundance,^144^ which may exhibit anti-inflammatory effects.^145^ However, changes in human gut microbiota after bariatric surgery are highly variable amongst individuals and its alterations in HF patients remain unclear.

Despite the debatable concept of the ‘obesity paradox’, which suggests a better prognosis in overweight/obese HF patients, *intentional* weight loss is beneficial in patients with established HF. Dietary intervention or exercise training in overweight or obese HFpEF patients improved exercise tolerance^146^ and cardiorespiratory fitness.^147^ A systematic review also suggests that weight loss intervention in HFpEF and HFrEF patients is promising, and may improve exercise capacity, NYHA classification and quality of life.^148^ In contrast, *unintentional* weight loss was associated with higher mortality and hospitalisation in both HFpEF^149^ and HFrEF patients.^150^ Unintentional weight loss is also associated with poor clinical prognosis and is indicative of cachexia,^151^ which is an independent risk factor for mortality in HFrEF patients. Owing to the potential benefits of intentional weight loss in HF patients, a recent consensus statement stated that weight loss should be a specific target in HFpEF patients; however, bariatric surgery is currently not specifically recommended for HF patients due to the lack of clinical trials.^152^

### 3.2 Available pharmacotherapies

#### 3.2.1 Glucagon-like peptide-1 receptor agonists

GLP-1 is a hormone secreted by the gastrointestinal tract in response to food ingestion to lower post-prandial blood glucose by stimulating insulin production and suppressing glucagon secretion.^153^ GLP-1RA is approved by the United States Food and Drug Administration for the treatment of type 2 diabetes mellitus and obesity. GLP-1RA administration delays gastric emptying, increases gastric volumes, reduces food intake and appetite, and enhances fullness and satiety, consequently promoting weight loss.^154^ Treatment with GLP-1RA reduced the occurrence of cardiovascular events in obese or overweight participants without diabetes.^155^ A recent meta-analysis revealed that GLP-1RA reduced HF hospitalisation, all-cause mortality, and atherosclerotic events in type 2 diabetic patients without HF.^156^ Therefore, GLP-1RA may be beneficial in preventing the onset of HF in both obese/overweight and type 2 diabetes patients with no history of cardiovascular disease.

The effects of GLP-1RA on the biodiversity of gut microbiome and cardiac metabolism are equivocal. For example, liraglutide treatment (1.8 mg/day for 12 weeks) has either no effect^157^ or increased^158^ the gut microbiome diversity by increasing the abundance of Bacteroidetes, Probacteria, and Bacilli in patients with type 2 diabetes. In terms of heart function, albiglutide treatment reduced infarct size, improved cardiac function, and increased myocardial glucose uptake in an ischaemic-reperfusion injury rodent model.^159^ In contrast, an overnight intra-venous infusion of GLP-1 (1.5 pmol/kg/min) increased myocardial glucose uptake under resting conditions only in lean participants, but not in type 2 diabetic patients.^160^ Similarly, 12-week albiglutide treatment in NYHA class II or III HFrEF patients did not affect myocardial glucose utilisation as determined by PET imaging.^161^ Therefore, further studies are required to clarify the gut microbiome and cardiometabolic effects of GLP-1 in patients with metabolic syndrome and HF.

Although GLP-1RA may be beneficial for the prevention of HF in diabetic and obese patients, the use of GLP-1RA for HF treatment remains uncertain. GLP-1RA treatment reduced the risk of HF hospitalisation in patients with no history of HF, but this outcome was not observed in patients with a history of HF.^162^ GLP-1RA treatment also did not improve left ventricular ejection fraction in HF patients with or without diabetes.^163^ GLP-1RA treatment in HF patients without diabetes has yielded conflicting results. In a recent STEP-HFpEF trial, once-weekly semaglutide treatment in obese, non-diabetic HFpEF patients for 52 weeks reduced HF-related symptoms, body weight, and NT-proBNP levels as well as improved physical and exercise function and 6-min walk distance.^164,165^ However, the effects of semaglutide on cardiac hemodynamics were not assessed in this study. In a small study involving 12 HFrEF patients without diabetes, 5-week infusion of GLP-1 improved left ventricular ejection fraction and 6-min walk distance when compared to patients receiving standard therapy alone.^166^ In contrast, 48-h GLP-1 infusion had no effect on ejection fraction and BNP level in 20 non-diabetic patients with ischaemic heart disease.^167^ Importantly, GLP-1RAs are known to elevate heart rate, and increased heart rate was associated with increased risk of adverse outcomes in HFrEF patients.^168^ Therefore, careful consideration regarding the presence of comorbidities, types of HF, treatment dose and duration, and further study on the safety of GLP-1RA as a potential cardioprotective strategy in HF are warranted.

#### 3.2.2 Sodium–glucose cotransport-2 inhibitors

SGLT2i was originally developed as an oral anti-hyperglycaemic medication for diabetes. Inhibition of sodium–glucose co-transporters in the proximal tubule of the kidney reduces glucose and sodium reuptake thereby lowering blood glucose. The EMPA-REG OUTCOME trial revealed that empagliflozin reduced cardiovascular mortality, hospitalisation for HF, and death from any cause in patients with type 2 diabetes.^169^ A meta-analysis of 13 large clinical trials involving over 90 000 participants confirmed these results in both HFpEF and HFrEF patients with and without diabetes.^170^ A recent study revealed that dapagliflozin treatment in HF with mildly reduced ejection fraction or HFpEF patients caused an initial estimated glomerular filtration rate decline of >10%; however, this was not associated with the subsequent risk of cardiovascular or kidney events.^171^ SGLT2i has now emerged as a new ‘pillar’ for HF treatment. In this section, we will discuss the potential mechanism of SGLT2i in HF with a focus on myocardial energetics and metabolic aspects. Other potential mechanisms such as natriuresis, sodium–hydrogen exchange inhibition, and reduction of inflammation have been proposed, however, these are out of the scope of this review.^172^

Although it remains controversial whether cardiomyocytes express SGLT2,^173,174^ SGLT2i therapy may exert direct cardiac effects and reverse ventricular remodelling in HF. SGLT2i therapy improved diastolic function in HFpEF patients with diabetes,^175^ and normalized passive force and diastolic tension in cardiomyocytes and ventricular trabeculae isolated from end-stage HF patients.^176^ SGLT2i therapy also reduced left ventricular mass and NT-proBNP level, and improved left ventricular function in diabetic patients with left ventricular hypertrophy,^177^ coronary artery disease,^178^ non-obstructive hypertrophic cardiomyopathy,^179^ and in non-diabetic HFrEF patients.^180,181^ There is compelling evidence that SGLT2i exerts direct beneficial cardioprotective effects in both HFpEF and HFrEF patients regardless of diabetes status.

SGLT2i therapy is often associated with an increase in circulating ketone levels in HF.^182^ Therefore, it has been proposed that SGLT2i improves myocardial energetics by increasing ketone body metabolism. Human data on the effects of SGLT2i on myocardial energetics are scarce and limited to two studies in diabetic patients without HF.^183,184^ In a recent EMPA-VISION trial, Hundertmark *et al.* found that empagliflozin treatment for 12 weeks (10 mg once daily) did not improve myocardial PCr/ATP in patients with HFrEF or HFpEF, and no change in serum β-hydroxybutyrate level was observed^12^; however, ketonaemia was frequently seen in other clinical studies.^182,185^ In contrast, animal studies have shown increased cardiac ATP content following SGLT2i treatment with either increased^185^ or decreased ketone utilisation.^186^ Pre-clinical studies have yielded conflicting results. SGLT2i administration increased ketone body utilisation in a non-diabetic HF porcine model as indicated by increased myocardial ketone body uptake,^185^ but not in a diabetic *db/db* mouse model.^186^ In *ex vivo* hearts isolated from pressure-overload induced HF mice, the cardioprotective effects of SGLT2i were sustained even in the absence of changes in ketone bodies.^187^ These data may suggest that improved energetics is not the key mechanistic benefit underlying ketonaemia because SGLT2i exerts direct benefits on isolated hearts without changing ketone levels in HF. Therefore, signalling pathways downstream of ketonaemia may be the important mediators.

Elevated ketones are reproducibly seen with SGLT2i therapy,^182,185^ and ketogenesis is a hallmark of nutrient deprivation. Instead of serving as a fuel for ATP production,^188^ an increase in circulating ketone bodies following SGLT2i therapy may represent a stimulus that promotes nutrient deprivation signalling and autophagy. SGLT2i up-regulated sirtuins and improved cardiac function by enhancing autophagic flux in a murine doxorubicin-induced cardiomyopathy model.^189^ AMPK, which acts as an energy sensor, inhibits several ATP-consuming biosynthetic pathways when ATP level is low to promote energy restoration.^190^ SGLT2i therapy simultaneously increases AMPK phosphorylation (activation)^185^ and decreases mTOR phosphorylation,^191^ leading to improved heart function in HF. Activation of mTOR in response to growth factors and nutrients promotes cell growth and proliferation. Therefore, suppression of mTOR following SGLT2i therapy reduces cellular biogenesis, thereby conserving energy. This data may suggest that SGLT2i induces a nutrient deprived state with simultaneous up-regulation of AMPK and sirtuins, and decreases activation of mTOR, which ultimately leads to reduced cellular stress and promotes cell survival.

### 3.3 Aspirational therapies

This section will focus on aspirational therapies for cardiac metabolism, including gene therapy, RNA-based therapeutics, and small-molecule activators/inhibitors. Metabolic profiling of the myocardium in the context of HFrEF and to a lesser extent HFpEF has provided some clues to promising therapeutic targets. The ever-more impaired myocardial oxidative capacity seen with progression of HFrEF leads to a switch of substrate usage along a gradient of substrate oxygen requirement, from fatty acids (lowest P/O ratio) to glucose (highest P/O ratio). The energetic perturbations in HFpEF are more varied compared to those in HFrEF due to the heterogeneity of phenotypes within the HFpEF spectrum. For example, HFpEF patients with metabolic syndrome are associated with insulin-resistant, which affects the metabolism of glucose.^192^ In these cases, other substrates that are less costly in terms of oxygen consumption yet not dependent on insulin sensitivity, e.g. ketone bodies, become important substrates. However, recent data have suggested commonality of changes in myocardial fatty acid usage in HFrEF and HFpEF, despite a significant difference in prevalence of diabetes.^23^ This work also suggested a decreased capacity of human HFpEF hearts to use alternative fuels (glucose, ketones, and BCAAs), consistent with a state of metabolic inflexibility. There was a consistent perturbation of metabolites in HFpEF hearts despite intra-group variability in obesity and other comorbidities, suggesting a common metabolic profile in HFpEF despite phenotypic variability.

It is worth considering whether the gate-keepers of metabolic regulation—transporters and enzymes—are amenable to gene therapy in HF. Decreased FAO in HFrEF results from decreased expression of the sarcolemmal fatty acid transporter CD36, along with decreased expression of CPT-1 and medium-chain acyl-coenzyme A dehydrogenase.^23^ However, an important caveat is the specific metabolic milieu in which the HF has developed. Specifically, in the context of obesity related HF, CD36 expression may be increased and related to the development of lipotoxicity.^193^ As such, interventions focused on augmenting fatty acid transport or metabolism in HF may require a personalized approach to avoid the exacerbation of lipotoxicity. Furthermore, glycerol-3-phosphate dehydrogenase (GPD) represents the inter-section point between glycolysis and fatty acid metabolism. The expression of GPD2 is increased in the presence of ischaemia but its deficiency exacerbates cardiac dysfunction post-myocardial infarction.^194^ Recently, it has been demonstrated that miR210 targets the 3'UTR of the GPD gene and that a miR210 mimic provides potential protective effect in the setting of ischaemia.^195^ This observation supports a strategy to augment cardiac function via regulators of cardiac metabolism.

Regarding HDACs as targets in hypertrophic cardiomyopathy and HF, it was previously demonstrated that ITF2357 (givinostat), a clinical-stage inhibitor of HDAC catalytic activity, mitigated diastolic dysfunction in two distinct murine models of diastolic dysfunction with preserved EF.^196^ ITF2357 attenuated diastolic dysfunction due to hypertension in Dahl salt-sensitive rats and mitigated aging-induced diastolic dysfunction in normotensive mice.^196^ HDAC inhibitor-mediated efficacy was not due to lowering blood pressure or inhibiting cellular and molecular events commonly associated with diastolic dysfunction, including cardiac fibrosis, cardiac hypertrophy, or changes in cardiac titin and myosin isoform expression. These authors concluded that the mechanism was rather a previously unrecognized, myocyte-autonomous mechanism for diastolic dysfunction. Other tractable HDAC targets include class III HDACs/sirtuins, using NAD^+^ repletion. Indeed, several groups have demonstrated that NAD^+^ repletion can reverse HFpEF in murine models, using nicotinamide (NAM)^52^ or nicotinamide riboside,^49^ and recent clinical work demonstrated NAD^+^ repletion was effective at suppressing inflammation in PBMCs in HF patients.^197^ Our group are currently undertaking two clinical trials of NAD^+^ repletion in HFpEF: CardioNAD (ACTRN12622000340730) and pEFNAD (ACTRN12622000372785).

A recent exciting development was the demonstration of blood pressure reduction in treatment naïve essential hypertension patients using pre-biotic supplementation enriched with SCFAs—this chemically-modified fibre delivers high levels of SCFAs to the large intestine via gut fermentation.^107^ In this study, led by F.Z.M., a 3-week pre-biotic acetylated and HAMSAB supplementation led to a clinically relevant (6.1 mmHg) reduction in 24-h systolic blood pressure independent of age, sex, and BMI without any adverse side effects. HAMSAB increased SCFAs acetate and butyrate levels, shifted the microbial ecosystem, and expanded the prevalence of SCFA producers. This study extended the observation of lowered blood pressure by SCFAs in experimental models. Remarkably, the reduction in systolic blood pressure achieved by HAMSAB was equivalent to that achieved by conventional anti-hypertensive mono-treatment.^198^ However, SCFAs have a host of beneficial effects beyond blood pressure reduction that target several of the current gaps in treatment for HF, especially HFpEF and particularly Stage A and B HF. For example, SCFAs provide beneficial metabolic and anti-inflammatory effects and in experimental models prevent cardiac hypertrophy, cardiorenal fibrosis, and HF.^106^ Promoting SCFA producing microbiota could also be beneficial in HF, such as *Ruminococcus sp*. that has a lower prevalence in patients with HF.^93^

The metabolite TMAO, derived from metabolism of dietary phosphatidylcholine, is elevated in patients with adverse prognosis for HF. In a murine TAC model of HF, dietary withdrawal of TMAO as well as use of a gut microbe-targeted inhibitor of TMAO production attenuated adverse ventricular remodelling and improved cardiac function.^199^ This study is clinically relevant, as complete eradication of gut microbiota is not a desirable approach given the many beneficial components of the gut microbiome, whereas the non-lethal TMA lyase inhibitor iodomethylcholine selectively targets a non-critical functional component maintaining essential functions of the microbiome. Another approach to inhibit gut microbial TMA production is the use of a structural analogue of choline, 3,3-dimethyl-1-butanol (DMB), and was shown to inhibit TMA production from polymicrobial culture of human intestinal contents and human faeces.^200^ Thus, this approach seems eminently feasible in humans, but clinical trials are awaited. The proposed mechanisms of the different therapies are summarized in *Table 1*.

**Table 1:** Summary of metabolic strategies in heart failure

Therapy	Intervention	Effects
Preventative (Stage A, B HF)	Diet	Calorie restriction induced weight loss, improved myocardial efficiency, and lipid profiles.^129^ Time restriction reduced BMI, HbA1c level, total cholesterol, triglycerides, and improved diastolic function.^130^ Dietary restriction (i.e. Mediterranean diet) reduced cardiovascular events,^134,135^ improved BMI, waist circumference, blood pressure, insulin resistance, and lipid profile.^136,137^ Mediterranean diet was associated with enrichment of SCFA producers in the gut microbiota.^138^
	Bariatric surgery	Reduced systolic blood pressure and HbA1c level.^140^ Reduced myocardial fatty acid utilisation.^141^ Reversed cardiac remodelling and improved cardiac function.^142^ Reduced risk of all-cause mortality and hospitalisation in HF patients.^143^ Increased gut *F. prausnitzii* abundance,^144^ which may exhibit anti-inflammatory effects.^145^
	Exercise	Improved exercise tolerance.^146^ Improved cardiorespiratory fitness.^147^
	GLP-1RA	Delayed gastric emptying, increased gastric volumes, reduced food intake and appetite, and enhanced satiety.^154^
Pharmacotherapy (All stages, HFrEF and HFpEF)	GLP-1RA	Reduced cardiovascular events in obese or overweight participants without diabetes.^155^ Reduced HF hospitalisation, all-cause mortality, and atherosclerotic events in diabetic patients without HF.^156^ Reduced body weight, HF symptoms and NT-proBNP levels, improved physical, and exercise function in non-diabetic HFpEF patients.^164,165^ Not recommended for HFrEF due to its effect to elevate heart rate.^168^
	SGLT2i	Reduced HF hospitalisation and mortality.^169^ Improved diastolic function.^175^ Reduced left ventricular mass and NT-proBNP level, improved left ventricular function.^177–181^ Increased circulating ketone bodies.^182,185^ Up-regulated sirtuins and promoted autophagy.^189^ Activated AMPK and promoted energy restoration.^185^ Suppressed mTOR hence reduced cellular biogenesis and conserved energy.^191^
Aspirational	Epigenetic therapy	Apabetalone (BET protein inhibitor) reduced HF hospitalisation and cardiovascular death.^201^ miR210 targeting glycerol-3-phosphate dehydrogenase gene and protected against ischemic injury.^195^ CDR132L (miR132 inhibitor) reduced plasma miR-132 and NT-proBNP levels in chronic HF.^202^ Givinostat/ITF2357 (HDAC inhibitor) prevented left ventricular diastolic dysfunction.^196^
	NAD^+^ repletion	Nicotinamide riboside improved mitochondrial function and reversed HFpEF phenotype.^49^ Nicotinamide improved diastolic function, myocardial bioenergetics, cardiomyocyte relaxation, and passive stiffness.^52^ Ongoing clinical trials: HFpEF: CardioNAD (ACTRN12622000340730) and pEFNAD (ACTRN12622000372785).
	Pre-biotics/Gut microbiome	HAMSAB supplementation decreased systolic blood pressure, shifted gut microbial ecosystem, and increased SCFA producers.^107^ SCFAs provided anti-inflammatory effects, prevented cardiac hypertrophy, cardiorenal fibrosis, and HF.^106^ Iodomethylcholine (TMA lyase inhibitor) attenuated adverse ventricular remodelling and improved cardiac function.^199^ 3,3-Dimethyl-1-butanol inhibited TMA production and attenuated atherosclerosis.^200^

## 4. Future directions

Despite recent insights in the field of cardiac metabolism, several challenges remain in the development of novel clinical therapies for HF. The concept of impaired metabolic flexibility in the failing heart is derived from a wealth of literature generated mainly from animal studies, albeit with ‘snapshots’ from human samples. It is important to consider that cardiac metabolism is dynamic. Hence, data derived from human tissues do not necessarily reflect the dynamic changes in the heart especially in diseased states. For instance, Watson *et al.*^11^ has recently provided evidence that metabolic flexibility is preserved in HFrEF patients both at rest and during increased workload via direct measurement of arteriovenous metabolite gradients. Direct assessment of cardiac metabolic flux in HFpEF is still lacking.

Furthermore, factors such as different HF aetiologies, sex and age differences, disease progression, fasting vs. non-fasting state, ‘snapshots’ vs. fluxomic measurements, inferences derived based on gene expression, protein level or enzymatic activity, targeted vs. untargeted metabolomics, variations in sample preparation, and whether studies have been focused on discrete metabolic pathways or unbiased comprehensive capture of the metabolome could contribute to the variations of the findings in the literature. Therefore, future studies should focus on *in situ* dynamic assessment of cardiac fuel utilisation and systematically address these variabilities using a standardized approach would improve our understanding on HF disease progression and accelerate the development of tailored therapy.

Recent technological advancement has opened an exciting area of research that enables simultaneous measurement of the dynamic metabolic changes and alterations in structure and function in the heart using magnetic resonance imaging (MRI). For instance, ^31^Phosphorus (^31^P) magnetic resonance spectroscopy was used to determine the PCr/ATP ratio^203^ and the rate of ATP synthesis through creatine kinase flux^204^ in HF patients; whereas ^1^Proton (^1^H) was used to quantify myocardial creatine and triglyceride levels.^205^ Additionally, hyperpolarized ^13^carbon (^13^C), which employs magnetic resonance spectroscopy using stable isotope ^13^C to trace carbon and the downstream ^13^C-labelled metabolites, allows real-time measurement of metabolic flux and contribution of individual energy substrates to the TCA cycle in human hearts *in vivo*.^206^ However, several challenges such as the requirement of specialized hardware and highly trained personnel may hinder the application of cardiac metabolic MRI in clinical and research settings. Nevertheless, cardiac metabolic MRI offers opportunities for direct quantification of metabolic changes *in vivo* non-invasively and is a promising approach to facilitate diagnosis and evaluate therapeutic outcome.

Pre-clinical models are highly valuable in facilitating the understanding of the pathophysiology of HF. However, limitations such as static environmental conditions, the lack of genetic diversity, differences in cardiac structure (i.e. myosin heavy chain isoform),^207^ and substrate utilisation between rodent and human hearts should be considered when translating findings from animal studies to humans. The mouse heart has a higher glycolytic rate (18%) than human hearts (5%).^208^ Cardiac responses to pressure-overload induced by TAC surgery also differ in various mouse substrains.^209^ Furthermore, establishing a pre-clinical model that fully recapitulates clinical HF is challenging due to the complexity and heterogeneity of HF especially HFpEF. Nevertheless, pre-clinical models remain a robust tool to model HF disease progression and allow genetic modification for precise mechanistic studies. Translation of pre-clinical findings to humans can be improved by refining animal models that could better recapitulate relevant clinical HF phenotypes.

Epigenetic modulation offers a promising avenue for HF management. Although many epigenetic drugs were initially approved for cancer treatments, their potential for the treatment of cardiovascular diseases has now become increasingly recognized. In the BETonMACE trial, Apabetalone, a selective inhibitor of bromodomain and extra-terminal (BET) proteins, reduced HF hospitalisation and cardiovascular death in patients with type 2 diabetes and acute coronary syndrome.^201^ A Phase 1b clinical trial demonstrated that CDR132L (a miR-132 inhibitor) was safe and effectively reduced plasma miR-132 and NT-proBNP levels in patients with chronic HF.^202^ This subsequently led to an ongoing phase 2 clinical trial to investigate the efficacy and safety of CDR132L in HFrEF patients (HF-REVERT) (NTC05350969).^210^ Epigenetic therapies hold great promise as novel therapies for HF, therefore further safety and later phase clinical trials are warranted.

## 5. Conclusion

Several recent developments have opened opportunities for targeting metabolism in HF including establishment of robust pre-clinical models, rapidly evolving metabolomic technology, careful clinical phenotyping, improved recognition of early (Stages A and B) HF, access to human cardiac bio-samples (transcardiac arterio-venous samples and cardiac biopsy), and novel pharmacotherapies. The establishment of SGLT2i as a pillar of HF treatment has reframed perspectives on HF therapy including roles of nutrient deprivation signalling and autophagy. Emerging therapeutic targets with promise include histone deacetylation (directly with HDACi, or indirectly using NAD^+^ repletion), modulation of gut microbiome-derived mediators of hypertension and inflammation, and epigenetic modifiers including BET inhibitors and CDR132L. In 2017, a Consensus Statement^47^ lamented an unfulfilled aspiration to address cardiac bioenergetics in HF; we may be at the dawn of an era in which this aspiration is finally realized.

## Data Availability

No new data were generated or analysed in support of this research.
